# Assessing Nursing Students’ Self-Perceptions about Safe Medication Management: Design and Validation of a Tool, the NURSPeM

**DOI:** 10.3390/ijerph19084663

**Published:** 2022-04-12

**Authors:** Pilar Fuster-Linares, Cristina Alfonso-Arias, Alberto Gallart Fernández-Puebla, Encarna Rodríguez-Higueras, Silvia García-Mayor, Isabel Font-Jimenez, Mireia Llaurado-Serra

**Affiliations:** 1 Nursing Department, Universitat Internacional de Catalunya, 08195 Barcelona, Spain; calfonso@uic.es (C.A.-A.); agallart@uic.es (A.G.F.-P.); erodriguez@uic.es (E.R.-H.); 2 Nursing Department, Faculty of Health Sciences, University of Malaga, 29071 Malaga, Spain; sgmayor@uma.es; 3 Nursing Department, Universitat Rovira i Virgili, 43005 Tarragona, Spain; isabel.font@universidadeuropea.es

**Keywords:** medication management, nursing students, patient safety, self-perceptions

## Abstract

Objective: The objective was to design and validate a tool for assessing nursing students’ self-perceptions about safe medication management. Methods: A descriptive instrumental study was conducted involving construct definition, development of the tool, analysis of the content validity, and psychometric evaluation. Consensus regarding the content was obtained through a two-round Delphi process, and the resulting tool (the NURSPeM) was administered to nursing students to examine its internal consistency, temporal stability, and construct validity, the latter through exploratory factor analysis. Results: Thirteen experts participated in the Delphi process, which yielded a tool comprising two questionnaires: (1) Self-perceptions about safe medication management (27 items) and (2) the frequency and learning of drug-dose calculation (13 items). The tool’s psychometric properties were then examined based on responses from 559 nursing students. This analysis led to the elimination of three items from questionnaire 1, leaving a total of 24 items distributed across seven dimensions. All 13 items in questionnaire 2 were retained. Both questionnaires showed good internal consistency (Cronbach’s alpha = 0.894 and 0.893, respectively) and temporal stability (ICC = 0.894 and 0.846, respectively). Conclusions: The NURSPeM is a valid and reliable tool for assessing nursing students’ self-perceptions about safe medication management. It may be used to identify areas in which their training needs to be enhanced, and to evaluate the subsequent impact of new teaching initiatives.

## 1. Introduction 

The safe management of medication is one of the most widely studied tasks due to its direct impact on patient safety (World Health Organization 2017). Although there have been increased efforts in recent decades to promote safety and prevent medical errors, the figures remain high. In the USA, medical errors are the third leading cause of death [1], while in our country, Spain, a 2015 report on patient safety and strategies to improve it concluded that 47% of adverse events were medication-related, and of these, almost 60% were preventable (Ministerio de Sanidad, Servicios Sociales e Igualdad [Spanish Ministry of Health, Social Services and Equality]) [2].

In 2017, the World Health Organization (WHO) launched its third Global Patient Safety Challenge: Medication Without Harm, aimed at reducing avoidable medication-related harm by improving practices and preventing medication errors [3]). The WHO report identifies three key action areas: Medication safety in high-risk situations, safety in polypharmacy, and transition of care. According to the WHO, as many as four out of every ten patients are harmed while receiving primary and ambulatory care, although the impact of medication errors is greater in the case of hospital inpatients. Importantly, the majority of medication errors are due to human factors and are more likely to occur during administration [4,5,6] Globally, the cost associated with medication errors has been estimated at US$42 billion annually (World Health Organization 2017).

Medication management is a complex process involving different professionals (pharmacists, physicians, and nurses), and also, in some cases, the patient [7]. Each stage of the process requires knowledge of standard protocols, administration routes, and interactions, as well as skills related to dose calculation, contextualizing the patient, and correct administration [8]. In the hospital setting, nurses play a key role in this process as they are the last link in the drug therapy chain, insofar as it is they who are responsible for drug administration. It is therefore crucial that nurses acquire, during their initial training, the knowledge and critical thinking skills that will enable them to identify any type of error which may occur during the process of prescribing, dispensing, and administering medication [5,6,9,10,11].

## 2. Background

In the literature on the safe management of medication, some of the most widely studied issues are types of error in professional nursing practice and a description of the causes [12,13,14,15], assessment of dose calculation competence and medication administration skills among both registered nurses and students [16,17], and teaching strategies for improving nurses’ medication competence and awareness [18,19,20]. However, some authors have focused their attention on more intangible elements of the process, such as the nursing role in safe medication administration [21,22,23], clinical reasoning, decision making [10,23,24,25] and nurses’ thinking process during medication administration [26,27]. Rohde and Domm [8] regard clinical reasoning as the element that best reflects nursing competence, while Armstrong et al. [10] conclude that attitudes are a key factor, insofar as they influence nurses’ clinical decision making, and because nurses prioritize work importance based on their attitudes. This means that critical thinking and clinical reasoning must be part of nurses’ professional competence [8], and their decision-making process should consider the key aspects of error management (i.e., professional competence, dose calculation skills, and safety guidelines).

Given the implications for patient safety, it is crucial that nurses begin to develop these competences during their initial training. Importantly, research conducted with nursing students has found that medication errors are the second major block of adverse events reported, accounting for around 30% of the total [28]. Anxiety, fear, inexperience, distractions, and mathematical incompetence have been identified as the main causes of error [29,30]. Although multicenter studies of nursing students’ arithmetic skills suggest that they are able to perform simple calculations, such as the required oral drug dose or maximum dose, a significant number of students struggle with calculations that imply a higher level of conceptual knowledge [31]. Furthermore, this difficulty appears to persist among registered nurses [32,33]. It is worth noting here that although students will be exposed to drug administration while on clinical placement, they are not always active participants in the process [8], and they may remain unaware of the internal process of critical thinking and knowledge-based decision-making that registered nurses engage in so as to ensure safe medication management [34,35].

A number of survey tools have been developed to assess professional nurses’ perceptions of the causes of medication errors, exploring aspects related to the task, teamwork, communication, and the work environment [36,37,38], and perceived attitudes and skills in relation to [21,39]. However, these tools may not be suitable for use with nursing students, due to their different levels of competence. Although some instruments have been developed specifically for use with health sciences students, they do not consider all the aspects of safe medication management that are of relevance to nursing students, and they do not focus specifically on their self-perceived competence in this regard. For example, the H-PEPSS instrument was designed to assess trainee health professionals’ perceptions, upon entry to practice, about their overall learning experiences in relation to patient safety in general [40]. The instrument developed by Aggar and Dawson [41], while aimed at nursing students, only assesses their preparedness for oral medication administration in clinical practice. Finally, Caboral-Stevens, Ignacio, and Newberry [42] adapted a questionnaire to assess nursing undergraduates’ pharmacology knowledge and self-rated certainty, on which basis they estimated the risk of error.

As Armstrong et al. [21] concluded, an expanded assessment of nurses’ attitudes and perceived skills in safety practices is important for identifying strategies to achieve sustainable improvement with adverse medication events and other safety events. With specific regard to the medication errors made by nursing students while on clinical placement, a recent review by Stolic et al. [30] identified poor critical thinking skills and a lack of adequate supervision as important factors associated with these errors, leading the authors to highlight the need to improve students’ decision making, critical thinking skills, and clinical judgement so as to ensure safe patient care. With these issues in mind, and to fill the gap identified by different authors, the aim of the present study was to design and validate a tool for assessing nursing students’ self-perceptions of their knowledge, skills, and attitudes in relation to the safe management of medication. To our knowledge, there is currently no instrument, whether for professional or student nurses, that offers such a comprehensive evaluation of this phenomenon. In the present manuscript, we describe the development and validation of the NURSPeM (Nursing students’ Self-Perceptions about Medication management), a tool that explores both self-perceptions about safe medication management and the frequency and practice of drug-dose calculation during training. This tool was created as part of a larger project that recognized the multidimensional nature of safe medication management [43]) and sought to address various aspects of this issue among nursing students. In particular, we believe it is important to explore how often nursing students have the opportunity to practice their drug calculation skills while on clinical placement, and also to understand more about their self-perceived competence in medication management and administration.

## 3. Research Question

In accordance with the aforementioned aim, the research question was as follows: Can the NURSPeM be considered a valid instrument for assessing nursing students’ self-perceptions of their knowledge and skills in relation to the safe management of medication?

## 4. Study Design

The study involved four phases: Definition of the construct and its dimensions as the basis for developing an initial set of items; development of the tool; analysis of content validity; and analysis of psychometric properties, including a pilot study and content validation with students from three different universities. The study was carried out from 2017 to 2019.

## 5. Method

In accordance with the recommendations for evaluating the quality of instruments measuring self-perceptions proposed by [44], as well as the guidelines for questionnaire design and validation described by [45,46], the process of developing and validating the NURSPeM involved four phases, as described in the next subsections.

### 5.1. Phase 1: Defining the Construct ‘Self-Perceptions about Safe Medication Management’ and Its Dimensions

We began by searching the PubMed database to gain an overview of the literature regarding nurses’ perceptions and self-perceptions about medication management. The key terms that emerged from this initial search were “perceptions and critical thinking” related to “clinical competence”, “medication error and error prevention”, “patient safety”, and “health knowledge, attitudes, practice” “instrument or tool” in nurses and nursing students. These terms were then used to conduct a definitive search of the CINAHL, Pubmed, and Cochrane databases. Three main themes emerged from the review:

(1) Analysis of safe medication management and the reasons for medication errors suggests that a key aspect to be explored further is nurses’ thought processes and self-perceptions of their skills and knowledge [10,23,47,48,49].

(2) The thinking process and clinical reasoning during medication administration [8,10,26]. According to these authors, nurses engage in a thinking process in order to prevent errors and harm to patients and promote a therapeutic response, with the ultimate aim being to administer medication safely.

(3) Perceptions and self-perceptions about medication management are closely related to recognition of the risks inherent to the use of medication [3]. This risk is present throughout the process of medication management (prescription, dispensing, administration, and monitoring or follow-up). The elements perceived as being most important vary depending on which actors are involved in a given stage of the process. In the literature search, we focused on nursing professionals and students.

Analysis of the information extracted in relation to these three themes gave rise to four areas of content for the tool (Table 1).

Based on the literature review, and in order to obtain a comprehensive measure of the construct “self-perceptions about safe medication management”, we developed a set of 45 items related to these four areas of content. Each area was considered as a theoretical dimension of the construct in the next phase of the study so as to ensure that all of the content areas identified were considered. In addition, when formulating the proposed items, we ensured that they explored students’ self-perceptions about all aspects of their competence, defined as “the integration and activation of knowledge, standards, technical procedures, attitudes, and values [50]. In this way, we were able to operationalize the initial theoretical construct. 

### 5.2. Phase 2: Development of the Tool: Items and Dimensions

The potential dimensions and items of the tool were discussed in a series of meetings involving the entire research team, using both inductive and deductive methods. The team comprised ten nurses (90% women) aged between 31 and 55 years, of whom 80% had a doctorate in nursing. The background of team members covered the clinical, academic, and research fields, and together they had clinical, management, and research experience in relation to both patient safety and the evaluation of competences. Their task here was to evaluate whether the proposed items were (a) consistent with the dimension to which they were linked and (b) suitable in relation to the purpose of the tool. The team was also invited to add or modify items, if considered necessary, and as a result, a further eight items were proposed. Of the 53 items, 30 were considered suitable for assessing nursing students’ self-perceptions about safe medication management and 23 for exploring the frequency and learning of drug dose calculations. Among the latter items, 13 referred to how often they calculated doses and their usual practice, while the remaining 10 items were mathematical exercises designed to assess their skills. By separating these 23 items into two blocks, they could be used to assess both students’ self-perceived competence in dose calculation, as well as their actual ability (through exercises). However, as the 10 practical exercises will have to be adapted to the characteristics of different student groups (e.g., made easier or more difficult depending on the stage of training), they do not form part of the validation process and are not described in the present study.

Based on this clear differentiation of the 53 items, we generated a provisional assessment tool, the NURSPeM (Nursing students’ self-perceptions about medication management), comprising two questionnaires: (1) Self-perceptions about safe medication management, and (2) the frequency and learning of drug-dose calculation.

### 5.3. Phase 3: Content Validity

In order to reach a consensus about which items to include in the tool, we employed the Delphi technique, a widely used method for assessing content validity when developing a measurement instrument [46]. 

A total of 17 experts were contacted by email and invited to participate in the Delphi process. This initial email also explained the purpose of the study and what would be required of them if they agreed to participate. In order to be eligible for the expert panel, participants had to currently be a member of their hospital’s clinical safety committee and fulfill at least one of the following criteria: (a) At least 10 years’ clinical experience and an interest in the area under study, and (b) at least five years’ experience as a lecturer in nursing studies. Fifteen of the 17 experts contacted agreed to take part and responded in the first Delphi round. They were sent, via email, the list of 53 items drawn up by the research team in Phase 2 of the study and were asked to rate the importance of each item with regard to measuring the construct of interest (i.e., nursing students’ self-perceptions about safe medication management). Ratings were given on a 5-point Likert-type scale (ranging from 0 “not at all important” to 4 “extremely important”). Experts were also invited to suggest any additional items and/or to indicate any items that were unclear and, in their opinion, should be reformulated.

The responses to this first Delphi round were then discussed in a meeting involving all ten members of the research team (see Phase 2), the aim of which was to reach a consensus regarding any necessary changes to the proposed content of the tool. To this end, and based on the ratings of the expert panel, we calculated the item-content validity index (I-CVI) [51] for each item, retaining those with a value >0.80. Items were marked for potential elimination if they generated polarized ratings, that is, three or more experts gave a rating of 0 or 1, while three or more experts rated the same item as 3 or 4 [52]. Any suggested reformulations or additions by the expert panel were also noted and evaluated. The results of this analysis were then collated and sent as feedback (again by email) to the 15 experts for consideration in the second Delphi round. Specifically, the experts were sent an anonymized summary of the overall results (i.e., ratings by the panel as a whole), as well as a reminder of their own rating for each item. Any proposed additions and reformulated items were also indicated. Their task in this second round was to rate the revised list of items once more, taking into account the feedback received. They were asked to pay particular attention to the items that had generated polarized responses in round one. The results from this second Delphi round were then analyzed by the research team, once again calculating the I-CVI for each item and recording any suggested changes. 

The two rounds of the Delphi process took place over a period of two months, with up to four reminders being sent to panel members to ensure they submitted their responses. Two of the 15 experts did not submit responses in round two, and hence the final analysis was based on the full set of responses obtained from 13 experts, whose characteristics were as follows: 11 (85%) were female and 8 (61%) had at least five years’ experience as a lecturer in nursing studies; 12 (92.3%) were professional nurses and 1 (7.7%) was a physician-epidemiologist who chaired a hospital patient safety committee. 

The outcome of this process was as follows: Questionnaire 1 initially comprised 30 items referring to three dimensions (perceived knowledge, thinking process, and training), while Questionnaire 2 consisted of 13 items concerning the frequency and practice of drug dose calculation. The content validation process for Questionnaire 1 led to 11 items being eliminated. Seven new items were added, and six existing items were reformulated. One item was sub-divided into two so as to enable a more precise evaluation, and two items with an I-CVI < 0.80 were nonetheless retained as they referred to issues of key importance in patient safety guidelines (i.e., the influence of workload and tiredness, respectively, on safe medication management). Questionnaire 1 therefore included 27 items. The same procedure was followed for Questionnaire 2, and all 13 items yielded an I-CVI > 0.80. Only three items were reformulated. The response format for all items (on both questionnaires 1 and 2) was a 5-point Likert-type scale from 0 to 4.

The two questionnaires were then piloted with a sample of 18 participants: 15 final-year nursing undergraduates and three experts in patient safety (none of whom had participated in the Delphi process). Each of the 18 participants was asked to complete the two questionnaires and evaluate the following three aspects: The clarity of items (i.e., the extent to which they are readily understandable); the format of the tool (i.e., the suitability of dividing the tool into two questionnaires and using a Likert-type response format for items); and the time needed to complete it. Based on the results of this pilot test, minor changes were made to the wording of a number of items. It took between 12 and 14 min to complete the two questionnaires, which was considered adequate by all participants in this phase.

### 5.4. Phase 4: Psychometric Evaluation of the Tool

Construct validity was examined by conducting an exploratory factor analysis to identify the dimensions of the tool. Reliability was assessed by analyzing temporal stability and the internal consistency of items. In order to carry out this psychometric evaluation, the piloted tool was administered to a sample of nursing undergraduates from three Spanish universities (two public and one a private, not-for-profit institution). In all three universities, drug-dose calculation was part of the course syllabus in each year of the degree and was covered both in theoretical modules (clinical skills) and on clinical placements. Based on the literature, we considered that a minimum of 5–10 students per item were needed as respondents [46]. In order to achieve this sample size, we recruited consecutively, over a period of two months, all students who met the following two inclusion criteria. First, they had to have completed at least one clinical placement (laboratory practice or clinical simulation did not count in this respect). This aspect was considered essential in order to achieve a minimum degree of sample homogeneity, that is to say, all the nursing students had access to theoretical knowledge about medication management, and they also had practical experience in a real healthcare setting, which was necessary for responding to both questionnaires. Second, they volunteered to take part.

Nursing students from each of the three universities were first informed about the nature of the study by one of their teachers during a scheduled class. They were told that if they wished to participate, they should remain in the room at the end of class, at which point a member of the research team would come and provide further details and answer any queries they had. The teacher was the first to leave at the end of class and hence had no knowledge of which students stayed behind. Those who did and who expressed to the researcher a willingness to take part were given written information about the nature of the study and were required to sign informed consent before receiving the two questionnaires. There was no time limit for completing the questionnaires, although all students did so in less than 20 min. The researcher remained in the room throughout to ensure that all responses were individual, and also that there were no interruptions. Participating students were not required to give any information that might identify them, and upon completing the questionnaires, they deposited them in a ballot box before leaving the classroom, thus ensuring anonymity and confidentiality. Students were not rewarded in any way (e.g., through extra course credits) for their participation.

**Figure 1:**
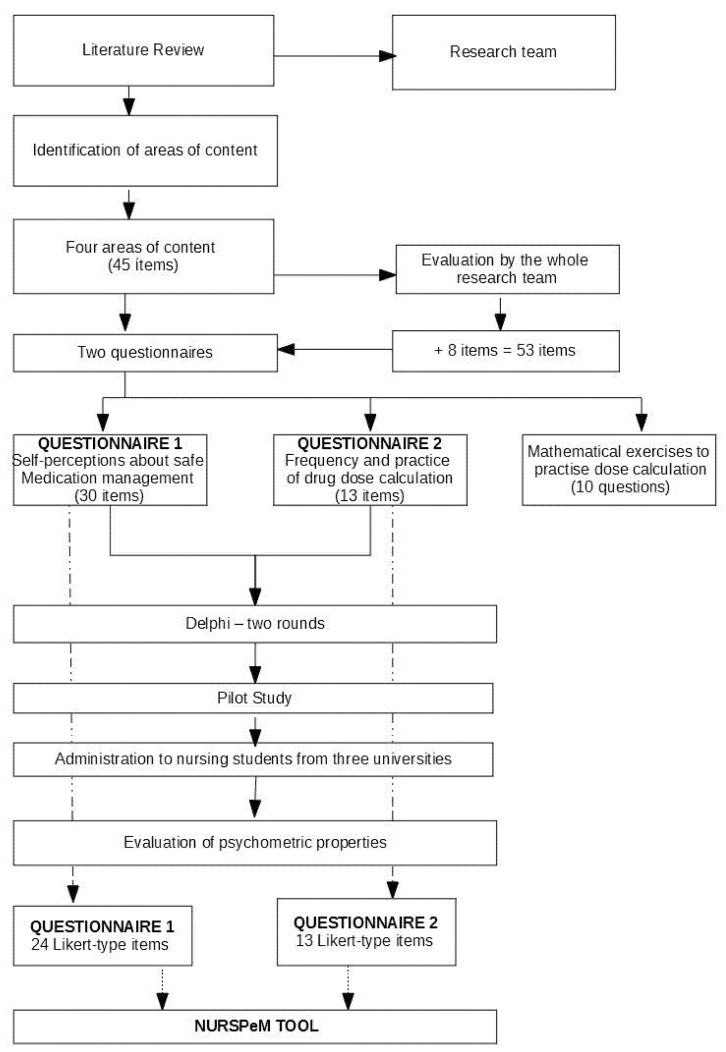
Flowchart showing the process of developing the NURSPeM.

Due to differences in the course syllabus of the three universities, eligible participants were currently in years two, three, or four of their degree program. Figure 1 shows the process followed in developing the NURSPeM.

## 6. Analysis

For the validation analysis, we considered only those questionnaires on which all items had been answered. The internal consistency of items was assessed by calculating Cronbach’s alpha. As a rule of thumb, alpha values are considered acceptable when they are above 0.70 and good when they are higher than 0.80 [46]. Temporal stability was analyzed by calculating intra-class correlation coefficients over a two-week interval, after first verifying the normality of the variables. In order to examine construct validity, we first standardized the variables to ensure they all had an equivalent weight in the analysis. We then conducted an exploratory factor analysis using the principal axis factor method and oblimin rotation [53]. The suitability of the exploratory factor analysis was verified by calculating the Kaiser–Meyer–Olkin (KMO) index of sampling adequacy (>0.80) and Bartlett’s test of sphericity (with *p* < 0.05). The number of factors to extract was determined based on the scree plot and eigenvalues > 1. Given the ordinal nature of variables, the analysis was based on the matrix of inter-item polychoric correlations.

For the descriptive analysis, we calculated means and standard deviations or absolute and relative frequencies, as appropriate. The level of significance was set at *p* < 0.05 for all analyses, which were performed using SPSS 21 for Windows (IBM, New York, NY, USA).

## 7. Ethics

The study was approved by the Research Ethics Committee of the lead researcher’s university (reference INF-2018-05), and permission was granted by the respective departments of nursing of the three participating universities. The confidentiality and anonymity of data were ensured at all times, and all participants gave their verbal and written consent. 

## 8. Results

A total of 581 questionnaires were returned (93.4% response rate), of which 22 were eliminated (due to two or more unanswered questions), leaving a sample for analysis of 559 questionnaires. Sixty-three of the nursing students completed the questionnaires again, two weeks after the initial administration, thus enabling us to examine temporal stability. Table 2 shows the characteristics of respondents in terms of age, gender, academic year, and clinical experience.

### 8.1. Construct Validity

***Questionnaire 1—Self-perceptions about safe medication management***: The exploratory factor analysis was conducted based on a total of 31 questions, because one of the 27 items was sub-divided into five statements. Although the model showed acceptable sampling adequacy, three of the items did not load on any of the questionnaire dimensions, and the communalities were very low (<0.200). These items (which referred to the importance of reporting adverse medication events, the need for double-check systems when preparing and administering high-risk drugs, and knowing which drugs require special attention with regard to patient safety) were therefore eliminated, and hence the revised questionnaire comprised 24 items.

This new model showed good sampling adequacy (KMO = 0.872), and Bartlett’s test of sphericity was significant (x^2^ = 6137.365; df = 378; *p* < 0.001). 

Item loadings on their theoretical dimension were above the established threshold of 0.35 [54] (see Supplementary Table S1). Examination of the scree plot and the results of oblimin rotation indicated a seven-factor model that explained 53.97% of the variance. The first factor explained 23.52% of the variance, the second 10.84%, the third 6.67%, up to the seventh that explained 2.03%. Table 3 shows the dimensions of Questionnaire 1.

***Questionnaire 2—Frequency and learning of drug dose calculation***: The exploratory factor analysis was conducted based on a total of 16 questions, because one of the 13 items was subdivided into five statements (one of these five statements was an open question and was therefore not considered in the factor analysis). As in the case of Questionnaire 1, the model showed good sampling adequacy (KMO = 0.876) and Bartlett’s test of sphericity was significant (x^2^ = 3581.596; df = 120; *p* < 0.001). Item loadings on each dimension were above the threshold of 0.35 (Hair 2018) (see Supplementary Table S1). The analysis indicated a three-factor model, although only two items loaded on the third factor. Given the recommendation that each factor should have at least 3–4 items [53], we forced a two-factor solution, which explained 44.33% of the variance (the first factor explained 36.11% and the second 8.22% of the total variance). The dimensions of Questionnaire 2 are shown in Table 3. 

The full list of items in questionnaires 1 and 2, along with their factor loadings, can be consulted in Supplementary Table S1. Supplementary Table S2 shows the inter-item correlations; note that, for both questionnaires, the items corresponding to each dimension are correlated with each other. The supplementary material also displays the final instrument.

### 8.2. Reliability Analysis

***Questionnaire 1—Self-perceptions about safe medication management***: Cronbach’s alpha for the whole questionnaire was 0.894. Table 3 shows the results of the validation analysis for the different dimensions identified. In general, the dimensions and the questionnaire as a whole show good internal consistency, although the value of Cronbach’s alpha for one dimension (0.649) is slightly below the acceptable threshold (0.70). 

The analysis of temporal stability in the subsample of 63 nursing students yielded an intra-class correlation coefficient of 0.894 (0.771–0.946), indicating high stability. The final version of Questionnaire 1 therefore comprised 24 items (see Figure 1).

***Questionnaire 2—Frequency and learning of drug dose calculation***: Cronbach’s alpha for the whole questionnaire was 0.893, and this was not improved by eliminating any of the items. Table 3 shows the results of the validation analysis for the different dimensions identified. The analysis of temporal stability in the subsample of 63 nursing students yielded an intra-class correlation coefficient of 0.846 (0.745–0.907), indicating high stability.

## 9. Discussion

The aim of the present study was to design and validate a tool (the NURSPeM) for assessing nursing students’ self-perceptions about safe medication management. This tool showed adequate psychometric properties. Both questionnaires had good internal consistency (alpha > 0.80), and test–retest reliability was also high (>0.70) [46]. However, one of the dimensions of Questionnaire 1 (referring to the importance of administering medication as prescribed) yielded an alpha coefficient slightly below the 0.70 threshold, and the items for this dimension were only moderately correlated with one another. Further investigation is warranted to elucidate possible reasons for this.

In the exploratory factor analysis of the tool, three of the initial items were eliminated as they did not load adequately on any factor and also yielded a low item-total correlation coefficient. These items referred to the importance of reporting adverse medication events, the need for double-check systems when preparing and administering high-risk drugs, and knowing which drugs require special attention with regard to patient safety. As to why these items did not reach the statistical thresholds for inclusion, in the case of error reporting, it could be due to how the students interpreted this item, that is to say, they may consider that error reporting goes beyond their responsibility, or, as some studies suggest [29,55], they may feel wary of communicating errors, given their status as students. It is worth noting here that nursing students in Spain are not required to report errors or adverse events while on clinical placement, as this is considered the responsibility of professional staff. Regarding the item about the need for double-check systems, the fact that these systems are not widely implemented in hospitals in our country may be a factor here. This raises the possibility that these two items may be susceptible to cultural influences [56], and thus they may need to be included if the questionnaire is adapted for use in other countries. Finally, and with respect to the item about knowing which drugs require special attention with regard to patient safety, we were surprised, given the importance of this issue, that this item did not meet the thresholds for inclusion. A possible explanation for this could be how the item was worded. It would therefore be useful to reformulate this item and examine in a new sample whether it met the criteria for inclusion in the questionnaire. 

### Development and Content of the Tool

The development of this tool addresses the need for an instrument able to assess more intangible aspects of safe medication management. This is important because research suggests that nurses’ perceived skills and attitudes in relation to safety practices play a role in medication errors [21]. Furthermore, and with specific regard to nursing students, a better understanding of their self-perceptions about this topic can help to identify areas in which their training needs to be enhanced [57,58]. It is worth noting here that the gap filled by the NURSPeM became evident during the process of developing and validating the tool, as it was not possible to analyze convergent or discriminant validity, due to the lack of other instruments designed to measure a similar or opposing construct. Although Márquez-Hernández et al. [39] recently reported the adaptation and validation for our cultural setting of a questionnaire to assess nurses’ knowledge, attitudes, and behavior, this tool was originally designed for the ICU setting and it does not address the range of issues considered by the NURSPeM.

The fact that our tool comprises two questionnaires reflects the complex and multidimensional nature of the construct under study, a point underlined by a recent concept analysis of medication competence [43]. In this respect, the application of the NURSPeM alongside the assessment of nurses’ actual knowledge and skills would enable a more comprehensive evaluation of their competence in relation to medication management. Furthermore, because nurses’ perceptions of their knowledge, skills, and attitudes in relation to safe medication management are likely to change with experience, the tool may be used to track nursing students’ development in relation to different aspects of the construct and to evaluate the effectiveness of interventions aimed at enhancing these areas of learning (Lee and Quinn 2019), including in real-life clinical practice. Various authors [16,18,19,43] have highlighted the importance of medication competence among both registered and student nurses, insofar as it is they, as the last link in the drug therapy chain, who are ultimately responsible for administration, where errors are most common [5,6]. However, attitudes towards safe medication management are also important and may be regarded as the first step in the process of ensuring safe practices [12,21]. In our view, this is particularly important in the case of nursing students, who are still at the learning stage and often find it difficult to transfer their theoretical knowledge to the clinical setting ([59]. Furthermore, although they have the opportunity during clinical placements to observe the administration and management of medication, they cannot see the internal process of critical thinking and knowledge-based decision making that is crucial to these practices [24,27]. As a result, they often focus solely on checking the five (or nine) rights of medication administration, the risk being that this then becomes a purely technical—rather than a reasoned—strategy [7,27]. It is important to remember that the clinical setting and care provision are inherently characterized by unique situations and complex problems and responding to them requires a reflective attitude among nurses [20]. In this sense, clinical practice offers nursing students an ideal opportunity to become aware of the importance of the decision-making and reflective process, and of how safe medication management depends on interdisciplinary collaboration among professionals [7,19]. Hence, these less-visible aspects need to be explored, evaluated, and made more explicit during clinical placements so that student nurses come to recognize the thinking and reflective process that underpins all aspects of everyday nursing practice [27,59]. A final point to consider is that the NURSPeM could also be used to explore how nursing students’ self-perceptions and awareness of the risks associated with medication administration and dose calculation [29] may be influenced by their experience in different clinical placement settings (hospital, primary care center, etc.). The link between risk awareness and safe medication management is one of the central themes addressed by the World Health Organization in its Patient Safety Curriculum Guide [60]

## 10. Future Research

In the Delphi study we conducted to reach an expert consensus on which items to include in the tool, we found that items referring to aspects involving a certain level of expertise or knowledge (e.g., accepting verbal prescriptions, knowing the potential toxicity of drugs) did not meet the criteria for inclusion. This shows how the importance ascribed to certain aspects is influenced by the level of expertise associated with them [61]. Consequently, although these issues are important for the safe management of medication, we do not consider them suitable for assessing this construct among nursing students. We are, however, in the process of validating a version of the NURSPeM for registered nurses, and this will enable a comparison of the two populations. The fact that the item referring to the importance of error reporting did not meet the criteria for inclusion in the final tool is perhaps an illustration of the gap that exists in this respect between student and professional nurses [29]. From the perspective of nurse education, it would be interesting to create error-reporting systems that students could use while on clinical placement to examine the effect this has on their risk awareness.

Further research into nursing students’ self-perceptions about safe medication management is needed to identify specific areas of knowledge, skills, and behavior that should be targeted during their training so as to lay the groundwork for an improved patient safety culture in the future. In this respect, the tool developed here could be used in studies aimed at comparing students’ self-perceptions with their actual real-world competences in this area.

## 11. Limitations

An important limitation of the present study is that the proposed tool only examines students’ self-perceptions and not their actual competences. That is to say, it assesses different aspects of what they think they know and do, which may or may not reflect what they actually think and do in clinical practice. Investigating the extent to which students’ self-perceptions and actual practice coincide would be an interesting topic for future research. A related limitation concerns the setting in which students completed the questionnaires. Although the presence of a researcher helped to ensure that responses were individual, and despite the fact that students were not rewarded in any way for their participation, we cannot rule out the possibility of influences such as social desirability bias, a problem that is inherent to studies of this kind. 

A further limitation to consider with regard to construct validity is that we only conducted an exploratory factor analysis. Although this approach is consistent with our research objective of designing a valid and reliable tool for assessing students’ self-perceptions regarding safe medication management, we acknowledge that confirmatory factor analysis is now needed to provide further information about the fit of our proposed model to the construct being measured. 

Furthermore, the present validation was conducted exclusively with students, and hence some aspects of the tool may need to be modified (e.g., rewording or eliminating certain items or adding new ones) before it could be applied to registered nurses.

## 12. Conclusions

The NURSPeM is a valid and reliable tool for assessing self-perceptions about safe medication management among nursing undergraduates, and it can therefore provide useful information about the less visible aspects of this process. An interesting task for future research would be to assess nursing students in different contexts and to examine whether there is a correlation between their clinical practice and their perceived and actual level of medication competence. The tool developed here could also be used to explore the transfer and application of learning once nursing students embark upon their professional careers, thus enabling further training initiatives to be targeted where they are most needed and helping to mitigate the theory-practice gap described in the literature.

## Figures and Tables

**Table 1:** General areas of content.

Areas of Content	Description of Content
Drug knowledge and safety	Knowledge about the drug, its dose(s), administration routes, and administration protocols and guidelines
Thinking process	Reflection on the process of drug administration (prior preparation, administration, checking the effect, contextualizing the patient), including information, communication, reading the physician’s orders, dose scheduling, and informing patients about their treatment.
Training in safe medication management	In-service training, updating knowledge, sources of information, awareness of responsibility in clinical practice
Medication administration skills	Dose calculation, dilutions, and volumes, material needed for drug administration, contextualizing the individual patient, frequency, and practice of drug-dose calculations.

**Table 2:** Characteristics of respondents.

	N (%)
University	
A	175 (31.3)
B	133 (23.8)
C	251 (44.9)
Female	448 (80.1)
Age [mean (SD)]	22.1 (4.8)
Experience working in health service (yes)	168 (30.1)
Currently working in the health service (yes)	66 (39.1)
Academic year *	
Second	228 (40.9)
Third	191 (34.2)
Fourth	139 (24.9)

* “One student did not provide information for academic year, and hence the total *n* = 558”.

**Table 3:** Summary of the results obtained for the dimensions of the two questionnaires.

Dimension	No. Items	% Variance	Cronbach’s Alpha
**Questionnaire 1**			**0.882**
Safety in drug prescription	4	23.525	0.828
Familiarity with pharmacological concepts	4	2.028	0.800
Relevance for professional practice	1 *	10.843	0.850
Factors associated with risk of error	3	6.668	0.789
Importance of administering medication as prescribed	4	2.490	0.649
Verifications prior to drug administration	3	5.452	0.762
Thinking process in relation to medication management	5	2.963	0.778
**Questionnaire 2**			**0.893**
Frequency of drug dose calculation	11	36.11	0.886
Learning drug dose calculation	2 **	8.22	0.772

* The item in this dimension is sub-divided into five questions. ** One of the two items comprising this dimension is sub-divided into four questions.

## Data Availability

All data will be accessible by contacting corresponding authors.

## Supplementary Materials

The following supporting information can be downloaded at: https://www.mdpi.com/article/10.3390/ijerph19084663/s1, Table S1. Loading factors from the exploratory factor analysis for the NURSPeM Questinnoaires 1 and 2; Table S2. Inter-item correlations of the questionnaires and the NURSPeM final questionnaire.
