# Supplementary material for: Assessing Nursing Students’ Self-Perceptions about Safe Medication Management: Design and Validation of a Tool, the NURSPeM
Source: Int J Environ Res Public Health. 2022 Apr 12;19(8):4663. doi: 10.3390/ijerph19084663 (PMC9028847; doi:10.3390/ijerph19084663)
Supplement: Supplementary file 1 [file ijerph-19-04663-s001.zip › ijerph-1608981-supplementary.pdf]

Table S1. Loading factors from the exploratory factor analysis for the NURSPeM.

Questionnaire 1: Self-perceptions about safe medication management

**Safety in drug prescription**								
		**Dimension 1**	**Dimension 2**	**Dimension 3**	**Dimension 4**	**Dimension 5**	**Dimension 6**	**Dimension 7**
**1**	How often do you know the most common dose of the drugs you administer to patients?	.791						
**2**	How often do you know the most common prescription of the drugs you administer to patients?	.740						
**3**	How often do you know the most common route of the drugs you administer to patients?	.653						
**4**	In general, do you feel confident about performing drug calculations?	.466						
**Familiarity with pharmacological concepts**								
**5**	To what extent are you familiar with the expected effects of the drugs that you administer to patients?		-.448					
**6**	To what extent are you familiar with the specific care or monitoring that a patient requires according to the drug you administer?		-.761					
**7**	To what extent are you familiar with the side effects of the drugs you administer to patients?		-.711					
**8**	To what extent are you familiar with the possible interactions that may exist between the drugs you administer to patients?		-.495					
**Relevance for professional practice**								
**9**	Rate the following statements according to the importance you believe they have for your professional practice:							
**9.1**	• Knowledge about drug effects			.556				
**9.2**	• Drug dose calculation			.569				
**9.3**	• Management of drug administration			.793				
**9.4**	• Drug interactions			.815				
**9.5**	• Required care of the patient during drug administration			.743				
**Factors associated with risk of error**								
**10**	Do you think that the way nurses organise their duties and manage medication has an influence on the likelihood of errors?				.482			

**11**	Do you think that workload can influence drug dose calculation and medication management?				.891			
**12**	Do you think that fatigue can influence drug dose calculation and medication management?				.886			
	**Importance of administering medications as prescribed**							
**13**	How important is it for you to administer the exact dose of the prescribed drug (without rounding off)?					-.620		
**14**	How important is it for you to check a patient’s drug prescription?					-.443		
**15**	How important is it for you to know how to calculate the dose of a prescribed drug?					.657		
**16**	To what extent do you think it is important to adhere to the prescribed frequency of the drugs you administer to patients?					-.427		
	**Verifications prior to drug administration**							
**17**	Do you check the patient’s identity before administering a drug?						-.798	
**18**	Do you check the six rights during drug preparation? *Six rights: right individual, right medication, right dose, right time, right route, right documentation.*						-.786	
**19**	How often do you check that the drug is in good condition (expiry date, stability, colour, appearance, etc.) before administering it?						-.410	
	**Thinking process in relation to medication management**							
**20**	Do you check for side effects related to the drugs you administer to patients?							-.645
**21**	Do you check the expected effect of the drugs you administer to patients?							-.645
**22**	Do you explain to the patient/family why a drug is being administered?							-.534
**23**	Do you explain to the patient/family the expected effects of the administered drug?							-.560
**24**	When planning and administering medication, do you take into account the other interventions (such as tests) that the patient undergoes?							-.741

Questionnaire 2: Frequency and learning of drug dose calculations

		**Dimension 1**	**Dimension 2**
**Frequency of drug dose calculation**			
**1**	How often do you calculate the dose of an oral medication?	0.578	
**2**	How often do you calculate the dose of a parenteral medication (IM, SC, IV)?	0.734	

**3**	How often do you calculate the dose of IV infusions?	0.811	
**4**	How often do you calculate the IV flow rate in ml/h?	0.791	
**5**	How often do you calculate the IV flow rate in drops/min?	0.717	
**6**	How often do you calculate the IV flow rate in microdrops/min?	0.593	
**7**	How often do you check drug calculations using a different mathematical method (reasoning, equation, etc.)?	0.460	
**8**	I feel confident about the drug calculations that I perform	0.432	
**9**	I ask someone else to check my drug calculations	0.355	
**10**	My drug calculation skills are assessed while on clinical placement	0.491	
**11**	I only maintain my drug calculation skills while on clinical placement	0.442	
**Learning drug dose calculation**			
**12**	I maintain my drug dose calculation skills by doing practice exercises in my own time		0.404
**13**	I studied drug calculation during my bachelor studies:		
	• By attending classes and clinical skills workshops		0.576
	• Through self-study with books		0.714
	• Through self-study with a computer		0.755
	• With another person		0.587
	• Another method. Please specify:		

Table S2. Inter-item correlations of the questionnaires.

Inter-item correlations - Questionnaire 1

	1	2	3	4	5	6	7	8	9,1	9,2	9,3	9,4	9,5	10	11	12	13	14	15	16	17	18	19	20	21	22	23	24
1	1,000																											
2	,744**	1,000																										
3	,626**	,592**	1,000																									
4	,470**	,482**	,431**	1,000																								
5	,480**	,481**	,511**	,433**	1,000																							
6	,516**	,492**	,475**	,435**	,518**	1,000																						
7	,473**	,490**	,435**	,363**	,598**	,558**	1,000																					
8	,432**	,441**	,361**	,343**	,471**	,555**	,590**	1,000																				
9,1	0,000	-0,008	0,018	0,016	,088*	0,020	0,026	0,021	1,000																			
9,2	-0,052	-0,075	-0,052	-0,054	-0,009	-0,004	-0,012	0,031	,431**	1,000																		
9,3	-0,025	-0,008	-0,023	-0,042	0,019	0,025	0,024	0,031	,476**	,477**	1,000																	
9,4	-0,019	-0,019	-0,075	-0,051	-0,025	-0,011	-0,009	-0,016	,509**	,414**	,580**	1,000																
9,5	-0,035	-0,053	-0,023	-0,051	0,000	0,031	-0,001	0,033	,467**	,395**	,569**	,561**	1,000															

10	0,018	0,021	0,038	-0,024	0,064	0,011	,114**	0,047	0,055	0,078	0,047	,087*	,114**	1,000														
11	0,064	,091*	,121**	-0,013	,106*	0,078	,087*	0,009	,097*	0,055	,110**	0,080	,102*	,469**	1,000													
12	0,018	0,053	,096*	-0,061	0,078	,093*	0,064	0,004	0,082	0,036	0,072	0,075	,090*	,473**	,784**	1,000												
13	-0,005	0,017	0,030	0,016	0,043	0,029	0,063	0,033	,186**	,247**	,237**	,164**	,214**	,165**	,184**	,167**	1,000											
14	,106*	0,064	,128**	,153**	,130**	,140**	,112**	,105*	,164**	,119**	,123**	,120**	,162**	,189**	,263**	,214**	,302**	1,000										
15	-0,005	0,008	0,076	0,058	0,025	0,059	0,037	0,016	,196**	,345**	,183**	,198**	,173**	,200**	,213**	,220**	,385**	,377**	1,000									
16	0,019	0,039	,084*	0,024	0,050	,129**	0,059	0,049	,197**	,198**	,176**	,139**	,160**	,157**	,212**	,237**	,326**	,246**	,335**	1,000								
17	,202**	,214**	,163**	,131**	,193**	,237**	,174**	,215**	,174**	,138**	,086*	0,072	,122**	0,041	,115**	,113**	,211**	,242**	,198**	,168**	1,000							
18	,246**	,240**	,201**	,190**	,223**	,244**	,155**	,161**	,136**	,151**	,088*	0,056	,138**	0,004	0,072	0,072	,114**	,172**	,156**	,125**	,638**	1,000						
19	,231**	,245**	,203**	,181**	,237**	,319**	,270**	,302**	,097*	0,078	0,053	0,024	,126**	0,014	0,067	0,065	,232**	,238**	,106*	,138**	,435**	,424**	1,000					
20	,325**	,361**	,323**	,295**	,318**	,396**	,359**	,310**	,188**	0,043	,106*	,115**	,181**	,121**	,115**	0,074	,194**	,232**	,185**	,139**	,402**	,409**	,417**	1,000				
21	,306**	,318**	,313**	,308**	,323**	,363**	,304**	,280**	,174**	0,064	,129**	,130**	,192**	,140**	,118**	0,077	,178**	,194**	,155**	,089*	,366**	,378**	,370**	,775**	1,000			
22	,314**	,280**	,344**	,264**	,323**	,296**	,281**	,246**	,132**	0,058	,096*	,129**	,128**	,095*	,110**	,116**	,245**	,226**	,167**	,146**	,313**	,312**	,224**	,395**	,396**	1,000		
23	,278**	,270**	,269**	,247**	,316**	,367**	,334**	,281**	0,076	0,058	,113**	,115**	,160**	,110**	,104*	0,076	,187**	,180**	,134**	,093*	,264**	,287**	,310**	,543**	,494**	,565**	1,000	
24	,323**	,313**	,340**	,287**	,285**	,306**	,280**	,254**	,194**	0,025	,093*	,101*	,181**	,122**	,187**	,151**	,230**	,246**	,188**	,174**	,386**	,380**	,320**	,530**	,486**	,495**	,563**	1,000

The inter-item correlation among the same dimension is highlighted in grey.

inter-item correlations - questionnaire 2

	1	2	3	4	5	6	7	8	9	10	11	12	13,1	13,2	13,3	13,4
1	1,000															
2	,669**	1,000														
3	,574**	,740**	1,000													
4	,459**	,612**	,776**	1,000												
5	,389**	,427**	,461**	,547**	1,000											
6	,359**	,358**	,385**	,397**	,714**	1,000										
7	,360**	,421**	,449**	,440**	,266**	,208**	1,000									
8	,425**	,466**	,496**	,506**	,290**	,248**	,498**	1,000								
9	,316**	,363**	,367**	,319**	,212**	,174**	,397**	,212**	1,000							
10	,456**	,503**	,499**	,507**	,291**	,241**	,435**	,547**	,384**	1,000						
11	,275**	,354**	,381**	,390**	,225**	,179**	,395**	,362**	,334**	,346**	1,000					
12	,337**	,332**	,364**	,321**	,223**	,245**	,432**	,379**	,300**	,417**	0,026	1,000				
13,1	,214**	,267**	,276**	,264**	,113**	,151**	,258**	,484**	,187**	,351**	,246**	,243**	1,000			
13,2	,231**	,217**	,234**	,214**	,154**	,215**	,255**	,316**	,195**	,313**	0,079	,444**	,387**	1,000		
13,3	,267**	,261**	,258**	,233**	,147**	,175**	,258**	,350**	,208**	,277**	,148**	,378**	,421**	,624**	1,000	
13,4	,274**	,356**	,314**	,302**	,129**	,142**	,307**	,324**	,333**	,380**	,197**	,330**	,415**	,417**	,507**	1,000

The inter-item correlation among the same dimension is highlighted in grey.

QUESTIONNAIRE ON THE SELF-PERCEPTION OF THE RELEVANCE OF SAFE DRUG MANAGEMENT (NURSPeM)

Questionnaire 1: Self-perceptions about safe medication management

The next questionnaire will explore your self-perception regarding drug management and administration. It has 24 questions grouped under 7 conceptual domains:

Domain 1: Safety in drug prescription
Domain 2: Familiarity with pharmacological concepts
Domain 3: Relevance for professional practice
Domain 4: Factors associated with risk of error
Domain 5: Importance of administering medication as prescribed
Domain 6: Verifications prior to drug administration
Domain 7: Thinking process in relation to medication management

Mark in the columns the value of the response according to your self-perception about the question asked.

Safety in drug prescription						
		**Never**	**Occasionally**	**Sometimes**	**Most of the times**	**Always**
**1**	How often do you know the most common dose of the drugs you administer to patients?	0	1	2	3	4
**2**	How often do you know the most common prescription of the drugs you administer to patients?	0	1	2	3	4
**3**	How often do you know the most common route of the drugs you administer to patients?	0	1	2	3	4
**4**	In general, do you feel confident about performing drug calculations?	0	1	2	3	4
Familiarity with pharmacological concepts						
**5**	To what extent are you familiar with the expected effects of the drugs that you administer to patients?	0	1	2	3	4
		**Not at all**	**Somewhat**	**Quite**	**Very much**	**Completely**
**6**	To what extent are you familiar with the specific care or monitoring that a patient requires according to the drug you administer?	0	1	2	3	4
**7**	To what extent are you familiar with the side effects of the drugs you administer to patients?	0	1	2	3	4
**8**	To what extent are you familiar with the possible interactions that may exist between the drugs you administer to patients?	0	1	2	3	4
**Relevance for professional practice**						

**9**	Rate the following statements according to the importance you believe they have for your professional practice:	**Not important at all**				**Very important**
**9.1**	• Knowledge about drug effects	0	1	2	3	4
**9.2**	• Drug dose calculation	0	1	2	3	4
**9.3**	• Management of drug administration	0	1	2	3	4
**9.4**	• Drug interactions	0	1	2	3	4
**9.5**	• Required care of the patient during drug administration	0	1	2	3	4
Factors associated with risk of error						
		**Not at all**	**Somewhat**	**Quite**	**Very much**	**Completely**
**10**	Do you think that the way nurses organise their duties and manage medication has an influence on the likelihood of errors?	0	1	2	3	4
**11**	Do you think that workload can influence drug dose calculation and medication management?	0	1	2	3	4
**12**	Do you think that fatigue can influence drug dose calculation and medication management?	0	1	2	3	4
Importance of administering medication as prescribed						
**13**	How important is it for you to administer the exact dose of the prescribed drug (without rounding off)?	0	1	2	3	4
**14**	How important is it for you to check a patient's drug prescription?	0	1	2	3	4
**15**	How important is it for you to know how to calculate the dose of a prescribed drug?	0	1	2	3	4
**16**	To what extent do you think it is important to adhere to the prescribed frequency of the drugs you administer to patients?	0	1	2	3	4
**Verifications prior to drug administration**						
		**Never**	**Sometimes**	**Often**	**Very Often**	**Always**
**17**	Do you check the patient's identity before administering a drug?	0	1	2	3	4
**18**	Do you check the six rights during drug preparation? *Six rights: right individual, right medication, right dose, right time, right route, right documentation.*	0	1	2	3	4
**19**	How often do you check that the drug is in good condition (expiry date, stability, colour, appearance, etc.) before administering it?	0	1	2	3	4
**Thinking process in relation to medication management**						
		**Never**	**Sometimes**	**Often**	**Very Often**	**Always**
**20**	Do you check for side effects related to the drugs you administer to patients?	0	1	2	3	4

**21**	Do you check the expected effect of the drugs you administer to patients?	0	1	2	3	4
**22**	Do you explain to the patient/family why a drug is being administered?	0	1	2	3	4
**23**	Do you explain to the patient/family the expected effects of the administered drug?	0	1	2	3	4
**24**	When planning and administering medication, do you take into account the other interventions (such as tests) that the patient undergoes?	0	1	2	3	4

*This questionnaire has been translated and culturally adapted into English, but the English version has not been validated.

Questionnaire 2: Frequency and learning of drug dose calculation

The following questions refer to calculation medication that you have performed during your placements. Please, answer according to the instructions below:

**Frequency of drug dose calculation**						
		**Never**	**Less than once /week**	**1-2 times a week**	**3-4 times a week**	**Daily**
**1**	How often do you calculate the dose of an oral medication?	0	1	2	3	4
**2**	How often do you calculate the dose of a parenteral medication (IM, SC, IV)?	0	1	2	3	4
**3**	How often do you calculate the dose of IV infusions?	0	1	2	3	4
**4**	How often do you calculate the IV flow rate in ml/h?	0	1	2	3	4
**5**	How often do you calculate the IV flow rate in drops/min?	0	1	2	3	4
**6**	How often do you calculate the IV flow rate in microdrops/min?	0	1	2	3	4
		**Never**	**Sometimes**	**Often**	**Very Often**	**Always**
**7**	How often do you check drug calculations using a different mathematical method (reasoning, equation, etc.)?	0	1	2	3	4
**8**	I feel confident about the drug calculations that I perform	0	1	2	3	4

**9**	I ask someone else to check my drug calculations	0	1	2	3	4
**10**	My drug calculation skills are assessed while on clinical placement	0	1	2	3	4
**11**	I only maintain my drug calculation skills while on clinical placement	0	1	2	3	4
**Learning drug dose calculation**						
**12**	I maintain my drug dose calculation skills by doing practice exercises in my own time	0	1	2	3	4
**13**	I studied drug calculation during my bachelor studies:	0	1	2	3	4
	• By attending classes and clinical skills workshops	0	1	2	3	4
	• Through self-study with books	0	1	2	3	4
	• Through self-study with a computer	0	1	2	3	4
	• With another person	0	1	2	3	4
	• Another method. Please specify:					

*This questionnaire has been translated and culturally adapted into English, but the English version has not been validated.

Tool developed and validated in Spanish by Llaurado-Serra M, Fuster P, Gallart A, Rodríguez-Higueras E / Nursing Department, Universitat Internacional de Catalunya, Spain. The present version in English has been translated and backtranslated and culturally adapted. However, it is not psicometrically validated. ©reproduced with the author's own permission.
